# The impact of serum thyroid-stimulation hormone levels on the outcome of hepatitis B virus related acute-on-chronic liver failure: an observational study

**DOI:** 10.1186/s12876-022-02406-7

**Published:** 2022-07-07

**Authors:** Jun-feng Chen, Wei-zhen Weng, Miao Huang, Xiao-hua Peng, Jing Zhang, Jing Xiong, Jian-rong He, Shao-quan Zhang, Hui-juan Cao, Bin Gao, Deng-na Lin, Juan Gao, Zhi-liang Gao, Bing-liang Lin

**Affiliations:** 1 Department of Infectious Diseases, Third Affiliated Hospital of Sun Yat-sen University, 600 Tianhe Road, Tianhe Area, Guangzhou, 510630 China; 2 Department of Nursing, Guangzhou Red Cross Hospital, Fourth Affiliated Hospital of Jinan University, Guangzhou, 510220 China; 3 Department of Gastroenterology, Seventh Affiliated Hospital of Sun Yat-sen University, Shenzhen, 518107 China; 4 Green Templeton College, University of Oxford, London, OX26HG UK; 5 Guangdong Provincial Key Laboratory of Liver Disease, The Third Affiliated Hospital of Sun Yat-sen University, Guangzhou, 510630 China; 6 Key Laboratory of Tropical Disease Control (Sun Yat-sen University), Ministry of Education, Guangzhou, 510080 Guangdong China

**Keywords:** Thyroid-stimulation hormone, Liver failure, Hepatitis B virus, Prognosis

## Abstract

**Background:**

Thyroid dysfunction has been reported in severe liver diseases. The aim of this study was to analyze the impact of serum thyroid-stimulation hormone (TSH) levels on the prognosis of patients with hepatitis B virus (HBV)-related acute-on-chronic liver failure (ACLF).

**Methods:**

This retrospective cohort study included 1,862 patients with HBV-related ACLF. Risk factors associated with 30-day and 90-day survival, hazard ratios (HRs), and 95% confidence intervals (CIs) for TSH were estimated using Cox proportional hazards regression. The Area Under the ROC curve (AUROC) analysis was carried out, and the cut-off values were calculated. After grouping by the cut-off value, survival was compared between the groups using the log-rank test. This study data is from the “Survival Cohort Study (SCS)”, which has been registered at ClinicalTrials.gov (NCT03992898).

**Results:**

Multivariate analysis indicated that an elevated TSH level was a highly significant predictor for 30-day survival (HR = 0.743, 95% CI: 0.629–0.878, *P* < 0.001) and 90-day survival (HR = 0.807, 95% CI: 0.717–0.909, *P* < 0.001). The AUROC of TSH level for 30-day and 90-day mortality were 0.655 and 0.620, respectively, with the same best cut-off values of 0.261 µIU/mL. Log-rank test showed that the group with higher TSH level had higher 30-day (78.5%, 95% CI: 76.1%-80.9% vs. 56.9%, 95% CI: 53.4%-60.4%; *P* < 0.001) and 90-day survival rate (61.5%, 95% CI: 58.6%-64.4% vs. 42.8%, 95% CI: 39.3%-46.3%; *P* < 0.001). Similar findings were observed in subgroups analysis. After adjusting for age and other risk factors, the higher level of TSH remained associated with 30-day survival (HR = 0.602, 95% CI: 0.502–0.721, *P* < 0.001) and 90-day survival (HR = 0.704, 95% CI, 0.609–0.814, *P* < 0.001).

**Conclusions:**

Serum TSH level significantly correlate with HBV-related ACLF patients’ survival and may be of value for predicting 30-day and 90-day survival of patients with HBV-related ACLF.

**Supplementary Information:**

The online version contains supplementary material available at 10.1186/s12876-022-02406-7.

## Background

Acute-on-chronic liver failure (ACLF) is an acute deterioration of known or unknown chronic liver diseases [1]. In Asia, ACLF is mainly caused by hepatitis B, and the mortality rate of patients who only receive comprehensive medical treatments is as high as 54% to 72% [1–3]. Liver transplantation can treat ACLF [4], however, the number of liver donors is limited. Therefore, liver transplantation is reserved for patients with the greatest needed. As such, it is important to identify prognostic indicators such that treatment can be optimized.

ACLF is a complicated condition, and can involve the dysfunction of multiple organs [5]. Prior studies have primarily focused on disorders of the liver, kidney, brain, heart, and lung and coagulation function in patients with ACLF [6–9]. Based on these studies, the Model for End-Stage Liver Disease (MELD) score [6], the European Association for the Study of the Liver (EASL)-Chronic Liver Failure (CLIF) Consortium Organ Failure (CLIF-C OF) score, CLIF-C ACLF score [7, 8], and Chinese Group on the Study of Severe Hepatitis B (COSSH) ACLF score [9] were developed to predict the outcomes of ACLF patients. However, these prognostic models do not include information of endocrine function, especially thyroid hormone levels. Thyroid hormones are essential for normal organ growth, development, and function [10]. Furthermore, they regulate the basal metabolic rate of all cells, including hepatocytes [11].

Thyroid dysfunction has been reported in most chronic illnesses, including severe liver diseases [12–15]. Thyroid stimulating hormone (TSH) is one of the important thyroid hormones, which play roles via the TSH receptor (TSHR). TSHR is a cell surface glycoprotein receptor. Several studies have demonstrated that TSHR was detected in many cells and tissues other than those in the thyroid, such as in hepatocytes, NK cells, erythrocytes, neuronal cells, et al. [16–20]. Wang X et al. [21] showed that TSH plays an essential role in mitochondrial oxidative stress in the liver via TSHR . Oxidative stress has been reported to be the collective pathophysiological mechanism of many liver diseases. In clinical trial, Wu D et al. [22] and Wu Y et al. [23] demonstrated that the serum level of TSH was negatively correlated with severity of hepatitis B virus (HBV)-related ACLF; patients with an increased TSH level have a higher survival rate. However, one of the studies [23] only included 75 patients with ACLF, and in the other study [22] the follow-up period was only 30 days. In addition, other studies have indicated that TSH level has no predictive value on the outcomes of patients with advanced liver diseases [15, 24, 25]. Therefore, whether the level of TSH can predict the outcome of hepatitis B virus (HBV)-related acute-on-chronic liver failure (ACLF) is controversial.

Thus, the purpose of this large cohort study was to determine the prognostic value of serum TSH levels in patients with HBV-related ACLF. Predictors of prognosis may assist in treatment planning for patients with HBV-related ACLF.

## Methods

### Patients

In this retrospective cohort study, patients with HBV-related ACLF were consecutively recruited from January 2010 to March 2018 at the Third Affiliated Hospital of Sun Yat-sen University, Guangzhou, China. This study data is from the “Survival Cohort Study (SCS)”, which has been registered at ClinicalTrials.gov (NCT03992898). This study included HBV-related ACLF patients older than 18 years (The specific inclusion criteria see our previous study [26]). Exclusion criteria were: 1) Primary thyroid diseases; 2) other 7 criteria (see our previous study [26]).

### Treatment, follow-up, outcomes and data collection

All patients were treated at the Third Affiliated Hospital of Sun Yat-sen University, Guangzhou, China with integrative medical therapy and were followed up for 90 days. The primary endpoint of the study was the 90-day transplantation-free survival rate. Data included demographic characteristics, biochemical indexes, complications of ACLF, etc. were recorded using the hospital information system (The specific protocol of treatment, follow-up and data collection see our previous study [26]).

### TSH measurement

TSH measurements were performed at the Central Laboratory of Third Affiliated Hospital of Sun Yat-sen University, using a chemiluminescence method and an automated system (Automatic Analyser Sysmex Centaur XP; SIEMENS Corporation, Munich, Germany). Reagents used and analysis protocols were based on SIEMENS AG guidelines. Serum TSH concentrations 0.55 to 4.78 µIU/mL were considered normal based on the manufacturer’s instructions.

### Statistical analysis

Continuous variables were described by median values and the 25th-75th percentiles (due to non-normally distributed continuous variables), and compared by Mann–Whitney U-test. Categorical variables were expressed as frequencies, and compared by chi-squared test. The Spearman correlation test was used to examine the association between TSH levels and other variables. Risk factors associated with 30-day and 90-day survival were examined using Cox proportional hazards models. Variables with values of *P* < 0.05 in univariate Cox regression analysis were included in the multivariate analysis using backward stepwise selection (Entry: 0.05, Removal: 0.1). Data were presented with hazard ratios (HRs) and 95% confidence intervals (CIs). The Area Under the ROC curve (AUROC) analysis of TSH for predicting 30-day and 90-day mortality was carried out, and the cut-off values were calculated. The Kaplan–Meier method was used to estimate the cumulative survival in relation to the cut-off values of TSH. Survival between the groups was compared using the log-rank test. HRs and 95% CIs for TSH cut-off values were estimated using Cox proportional hazards regression. We first adjusted for MELD, MELD with the addition of the Na level (MELD-Na), and Child-Turcotte-Pugh (CTP) scores and then further adjusted for risk factors that selected from multiple cox regression. All reported probability values were 2-tailed, and values of *P* < 0.05 were considered to indicate statistical significance. Statistical analyses were performed using SPSS version 22.0 software.

## Results

### Patient characteristics at baseline

**Fig. 1:**
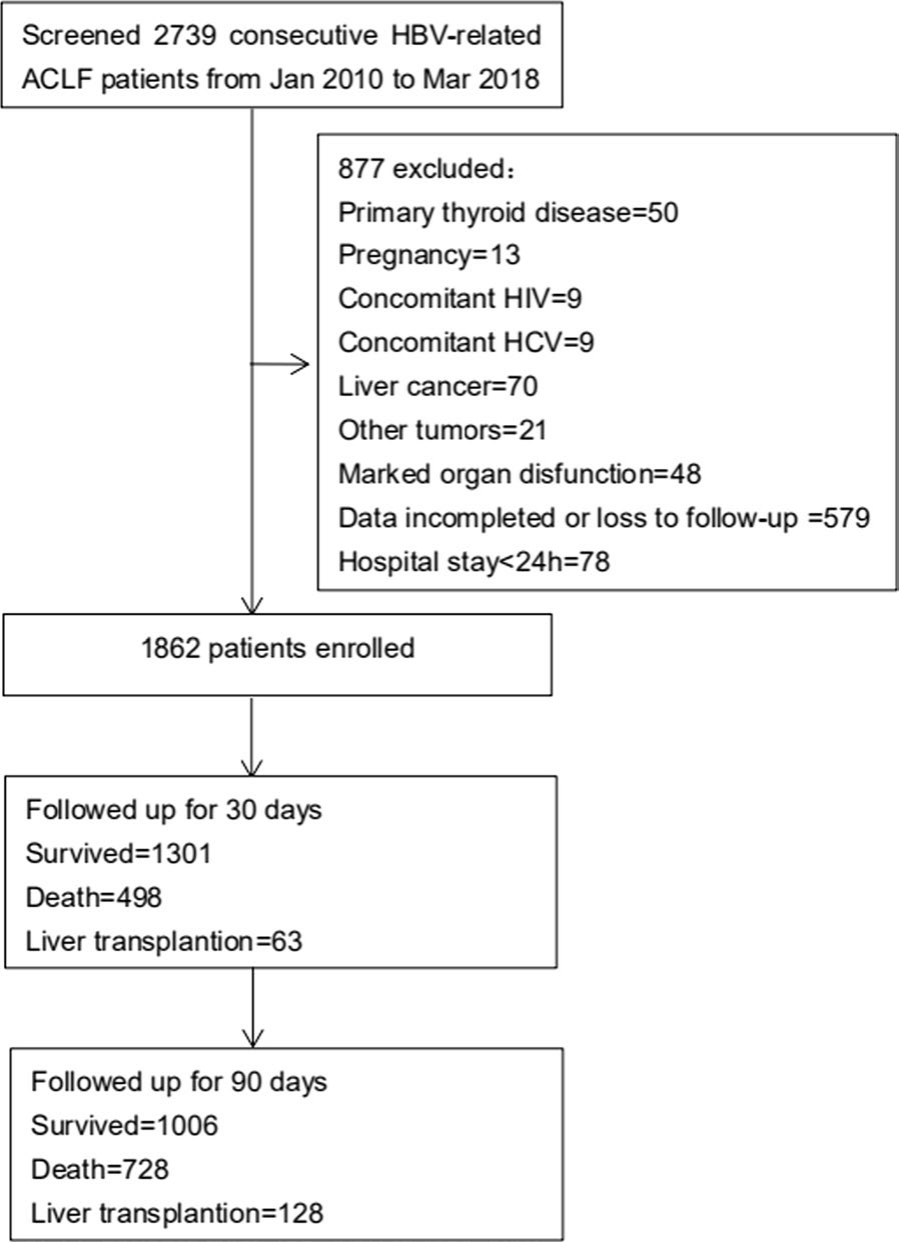
Study Profile. A total of 2739 (From January 2010 to March 2018) consecutive HBV-related ACLF patients were screened. Finally, 1862 patients were included. 1301 and 1006 patients were follow-up for 30 days and 90 days, respectively. ACLF, acute-on-chronic liver failure; HBV, hepatitis B virus; HCV, hepatitis C virus; HIV, human immunodeficiency virus

A total of 2,739 consecutive HBV-related ACLF patients were screened, and 1,862 patients were included in the analysis. 579 were excluded due to incomplete data or lost to follow-up (46 patients lost to follow-up, 131 patients lacked TSH information, and 402 patients lost to follow-up and lacked TSH information). The average follow-up period before lost to follow-up was 16.35 ± 12.73 days. OF the 1,862 patients 1,301 were alive at 30 days, and 1,006 were alive at 90 days (Fig. 1). The median TSH level of all patients was 0.389 µIU/mL (interquartile range [IQR]: 0.137–0.959). Baseline clinical and laboratory data of survival and non-survival patients are shown in Table 1.

**Table 1:** Patient demographics and clinical characteristics^†^

Characteristic	Survivor Group (for 90 days) (*n* = 1006)	Non-survivor Group (for 90 days) (*n* = 856)	*P* value
Age, y	41.0 (33.0,50.0)	49.0 (41.0, 58.0)	< 0.001
*Sex*			
Male	901 (89.6)	755 (88.2)	0.351
Female	105 (10.4)	101 (11.8)	
WBC (× 10^9^/L)	7.21 (5.60, 9.08)	7.85 (5.76, 10.59)	< 0.001
Hb (g/L)	124.0 (110.0,136.3)	120.0 (102.0, 134.0)	< 0.001
PLT (× 10^9^/L)	121.0 (88.0, 158.3)	102.0 (66.0,143.0)	< 0.001
ALT (U/L)	368.5 (118.8, 900.5)	322.0 (101.0, 848.0)	0.178
AST (U/L)	219.5 (112.8, 503.5)	257.0 (128.3, 609.5)	0.005
ALB (g/L)	33.0 (30.1, 35.7)	31.8 (29.0, 34.6)	< 0.001
GLB (g/L)	27.9 (23.9, 32.3)	28.5 (24.2, 33.3)	0.088
TBil (µmol/L)	338.7 (256.2, 438.9)	412.3 (307.1, 527.9)	< 0.001
INR	2.12 (1.83, 2.56)	2.82 (2.21, 3.70)	< 0.001
Na (mmol/L)	137.4 (135.0, 139.5)	136.0 (132.4, 138.7)	< 0.001
Cr (µmol/L)	69.0 (61.0, 79.2)	73.1 (62.0, 92.0)	< 0.001
AFP (ng/mL)	78.6 (29.5, 193.8)	29.8 (10.5, 95.1)	< 0.001
TSH (µIU/mL)	0.525 (0.188, 1.137)	0.265 (0.092, 0.720)	< 0.001
*HBeAg*			
Positive	364 (36.2)	233 (27.2)	< 0.001
Negative	642 (63.8)	623 (72.8)	
HBV DNA (IU/mL, log10)	5.12 (3.67, 6.53)	5.28 (3.70, 6.91)	0.191
MELD score	24 (22, 27)	29 (25, 33)	< 0.001
MELD-Na score	24.6 (22.2, 27.5)	30.5 (25.9, 36.2)	< 0.001
CTP score	10 (9,11)	11 (10, 12)	< 0.001
*Pre-existing chronic liver diseases*			
Chronic hepatitis	517 (51.4)	243 (28.4)	< 0.001
Cirrhosis	489 (48.6)	613 (71.6)	
*Ascite*			
None	418 (41.6)	312 (36.4)	< 0.001
Grade 1–2	490 (48.7)	392 (45.8)	
Grade 3	98 (9.7)	152 (17.8)	
*Hepatorenal Syndrome*			
No	997 (99.1)	790 (92.3)	< 0.001
Yes	9 (0.9)	66 (7.7)	
*Hepatic Encephalopathy*			
None	915 (91.0)	592 (69.2)	< 0.001
Grade 1–2	81 (8.1)	220 (25.7)	
Grade 3–4	10 (1.0)	44 (5.1)	
*Gastrointestinal Bleeding*			
No	1001 (99.5)	835 (97.5)	< 0.001
Yes	5 (0.5)	21 (2.5)	
*Infection*			
No Yes	361 (35.9) 645 (64.1)	168 (19.6) 688 (80.4)	< 0.001
*Antivirus Drug*			
None	43 (4.3)	96 (11.2)	< 0.001
LAM	31 (3.1)	29 (3.4)	
ADV	4 (0.4)	3 (0.4)	
ETV	831 (82.6)	658 (76.9)	
TDF	43 (4.3)	26 (3.0)	
Ldt	17 (1.7)	9 (1.1)	
Combination therapy	37 (3.7)	35 (4.1)	

^†^Demographics and clinical data were expressed as No. (%) or median and 25th-75th percentiles

*ADV* adefovir dipivoxil; *AFP* alpha fetal protein; *ALB* albumin; *ALT* alanine aminotransferase; *AST* glutamic-oxaloacetic transaminase; *Cr* serum creatinine; *CTP* Child-Turcotte-Pugh; *ETV* entecavir; *GLB* globulin; *Hb* hemoglobin; *HBeAg* hepatitis B e antigen; *HBV* hepatitis B virus; *INR* international normalized ratio; *LAM* lamivudine; *Ldt* telbivudine; *MELD* Model for End-Stage Liver Disease; *MELD-Na* Model for End-Stage Liver Disease with the addition of the Na level; *Na* serum sodiun; *PLT* platelet; *TBil* total bilirubin; *TDF* tenofovir; *TSH* thyroid-stimulation hormone; *WBC* white blood cell

### Distributions and associations with clinical variables of TSH

**Fig. 2:**
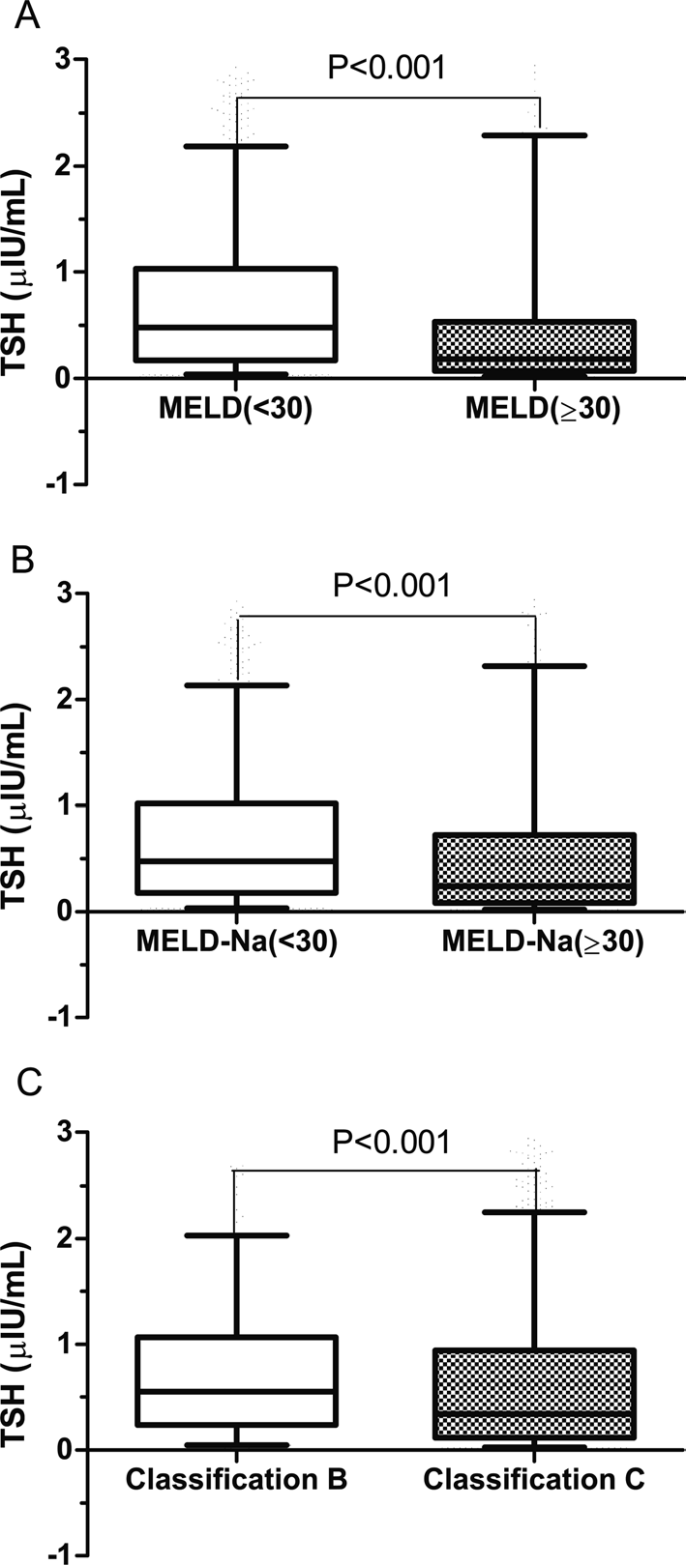
Comparison of serum TSH levels between different subgroups in patients with HBV-related ACLF. (**A**: MELD score < 30 vs. MELD score ≥ 30; **B**: MELD-Na score < 30 vs. MELD-Na score ≥ 30; **C**: CTP classification B vs. CTP classification C; Serum thyroid-stimulation hormone levels are expressed as the median and 5–95% percentile). ACLF, acute-on-chronic liver failure; CTP, Child-Turcotte-Pugh; HBV, hepatitis B virus; MELD, Model for End-Stage Liver Disease; TSH, thyroid-stimulation hormone

The TSH level was higher in patients with a MELD or MELD-Na score < 30 than in patients with MELD score ≥ 30 (0.48 µIU/mL, IQR: 0.17–1.03 vs. 0.18 µIU/mL, IQR: 0.07–0.53, *P* < 0.001) or MELD-Na ≥ 30 (0.48 µIU/mL, IQR: 0.18–1.02 vs. 0.24 µIU/mL, IQR: 0.08–0.73, *P* < 0.001). Moreover, TSH level was higher in patients with CTP classification B than in patients with CTP classification C patients (0.55 µIU/mL, IQR: 0.24–1.07 vs. 0.34 µIU/mL, IQR: 0.12–0.94, *P* < 0.001) (Fig. 2). (There were no CTP classification A patients in this study.). TSH level (as a continuous variable) was significantly correlated with MELD score (rs = -0.254, *P* < 0.001), MELD-Na score (rs = − 0.211, *P* < 0.001), and CTP score (rs = − 0.128, *P* < 0.001).

### Univariate and multivariate analyses of prognostic factors

Cox regression models indicated that TSH level was strongly associated with 30-day survival (HR = 0.521; 95% CI: 0.441–0.615, *P* < 0.001) and 90-day survival (HR = 0.648; 95% CI: 0.576–0.729, *P* < 0.001) (see Additional file 1: Table S1). In addition, age, sex, white blood cell (WBC) count, hemoglobin (Hb), platelet (PLT), alanine aminotransferase (ALT), aspartate aminotransferase (AST), serum albumin (ALB), total bilirubin (TBil), international normalized ratio (INR), serum sodium (Na), serum creatinine (Cr), alpha fetoprotein (AFP), HBV e antigen (HBeAg) status, HBV DNA level, hepatorenal syndrome (HRS), gastrointestinal bleeding (GB), hepatic encephalopathy (HE), infection, and pre-existing liver disease(chronic liver disease or cirrhosis) were significantly associated with 30-day survival; whereas, age, WBC count, Hb, PLT, AST, TBil, INR, Na, Cr, AFP, HBeAg status, HBV DNA level, ascites, HRS, GB, HE, infection, and pre-existing liver disease were significantly associated with 90-day survival.

Multivariate analysis indicated that an elevated TSH level was a highly significant predictor for 30-day survival (HR = 0.743; 95% CI: 0.629–0.878, *P* < 0.001) and 90-day survival (HR = 0.807; 95% CI: 0.717–0.909, *P* < 0.001), independent of age, WBC count, AST, and other risk factors (Table 2).

**Table 2:** Multivariate Cox proportional hazards regression analysis

Variable	30 days survival			90 days survival		
	HR	95%CI	*P*	HR	95%CI	*P*
Age,y	1.026	1.019–1.034	< 0.001	1.029	1.023–1.035	< 0.001
WBC (× 10^9^/L)	1.035	1.017–1.054	< 0.001	1.029	1.013–1.046	< 0.001
AST (U/L)	1.000	1.000–1.001	< 0.001	1.000	1.000–1.001	< 0.001
ALB (g/L)	0.969	0.949–0.990	0.003	–	–	–
TBil (µmol/L)	1.002	1.001–1.002	< 0.001	1.002	1.001–1.002	< 0.001
INR	1.064	1.046–1.082	< 0.001	1.060	1.045–1.076	< 0.001
Cr (µmol/L)	1.003	1.001–1.004	< 0.001	1.002	1.001–1.004	< 0.001
AFP (ng/mL)	0.998	0.997–0.999	< 0.001	0.999	0.998–0.999	< 0.001
HBV DNA (IU/mL, > 156,000 vs. < 156,000)	1.550	1.275–1.884	< 0.001	1.498	1.279–1.755	< 0.001
TSH(µIU/mL)	0.743	0.629–0.878	< 0.001	0.807	0.717–0.909	< 0.001
Pre-existing chronic liver diseases (Cirrhosis vs. Chronic hepatitis)	1.287	1.047–1.582	0.017	1.557	1.296–1.871	< 0.001
Hepatorenal Syndrome (Yes vs. No)	1.510	1.054–2.164	0.025	–	–	–
Hepatic Encephalopathy (Grade 3 vs. Grade 1–2 vs. None)	2.630	2.258–3.064	< 0.001	2.140	1.873–2.445	< 0.001
Gastrointestinal Bleeding (Yes vs. No)	–	–	–	1.636	1.052–2.543	0.029
Infection (Yes vs. No)	–	–	–	1.259	1.052–1.506	0.012

*AFP* alpha fetal protein; *ALB* albumin; *AST* glutamic-oxaloacetic transaminase; *CI* confidence interval; *Cr* serum creatinine; *HBV* hepatitis B virus; *HR* hazard ratio; *INR* international normalized ratio; *TBil* total bilirubin; *TSH* thyroid-stimulation hormone; *WBC* white blood cell

### AUROC analysis

**Fig. 3:**
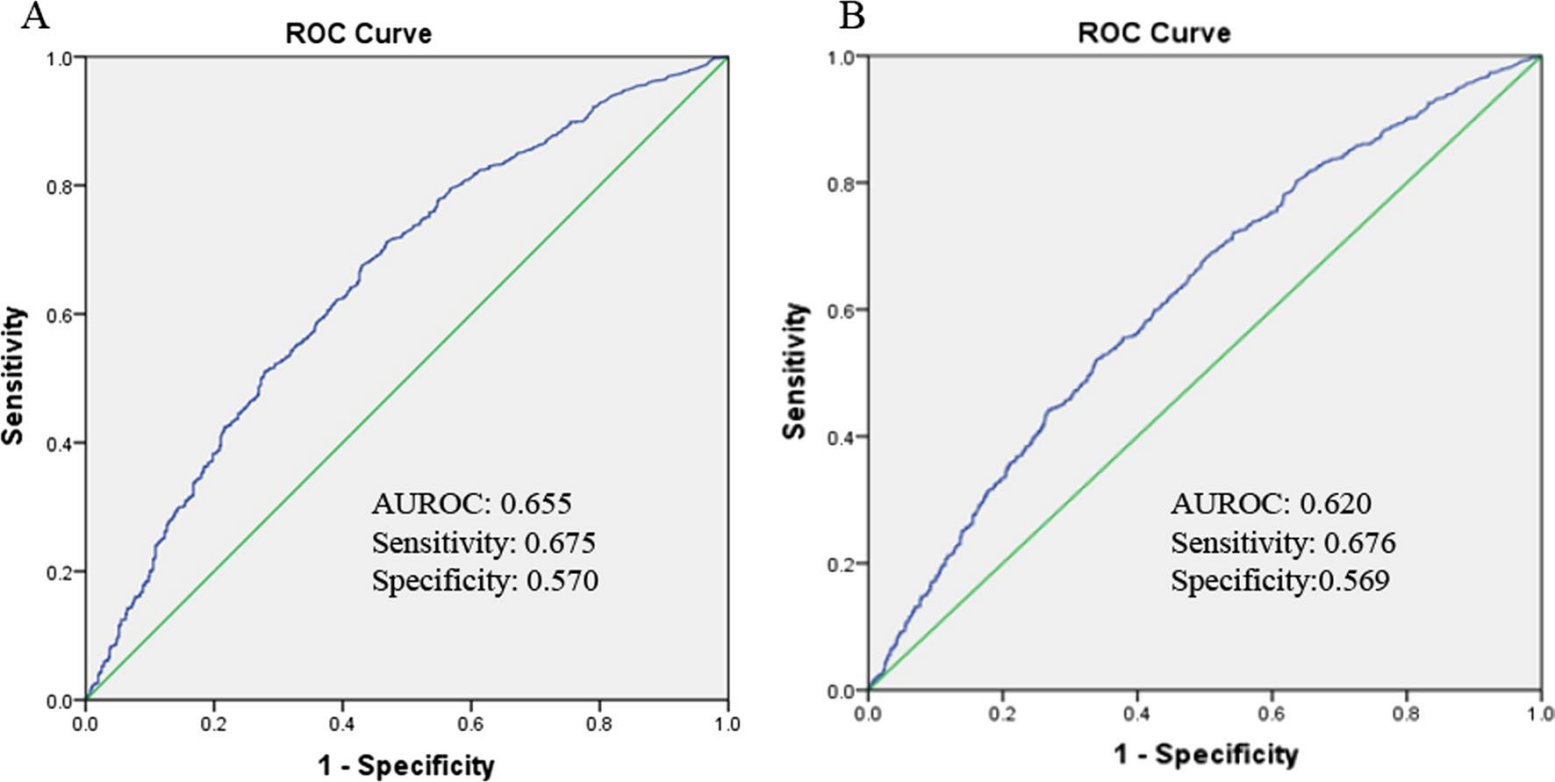
AUROC analysis of TSH level to predict mortality of HBV-related ACLF patients. (**A**: 30-day mortality; **B**: 90-day mortality). ACLF, acute-on-chronic liver failure; AUROC, Area Under the ROC curve; HBV, hepatitis B virus; TSH, thyroid-stimulation hormone

The AUROC of TSH level for predicting the 30-day and 90-day mortality were 0.655 (95%CI: 0.628–0.682, *P* < 0.001) and 0.620 (95%CI: 0.595–0.646, *P* < 0.001), respectively (Fig. 3). The sensitivity and specificity were 0.675, 0.676 and 0.570, 0.569, respectively. The best cut-off value of TSH for predicting the 30-day and 90-day mortality were the same, which was 0.261 uIU/mL.

### TSH levels and probability of survival

**Fig. 4:**
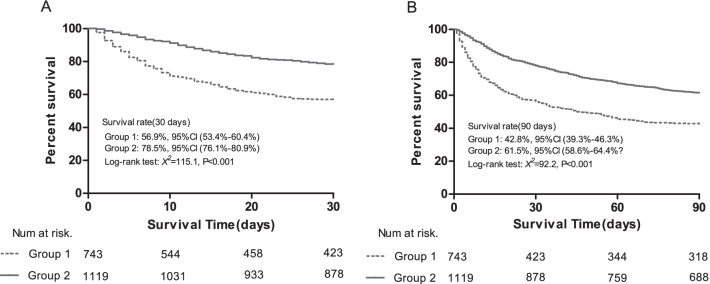
Kaplan–Meier curves of the entire HBV-related ACLF population according to serum TSH cut-off value. (**A**: 30-day survival, **B**: 90-day survival; Group 1: < 0.261 µIU/mL, Group 2: ≥ 0.261 µIU/mL). ACLF, acute-on-chronic liver failure; HBV, hepatitis B virus; TSH, thyroid-stimulation hormone

For Kaplan–Meier analysis, patients were categorized into 2 groups based on TSH cut-off value: Group 1, TSH < 0.261 uIU/mL; Group 2, TSH ≥ 0.261 uIU/mL. Kaplan–Meier estimates for 30-day and 90-day survival according to TSH cut-off value were shown in Fig. 4. Elevated TSH level was significantly associated with a higher 30-day (Group 1: 56.9%, 95% CI: 53.4%-60.4%; Group 2: 78.5%, 95% CI: 76.1%-80.9%; log-rank test *P* < 0.001) and 90-day survival rate (Group 1: 42.8%, 95% CI: 39.3%-46.3%; Group 2: 61.5%, 95% CI: 58.6%-64.4%; log-rank test *P* < 0.001).

Patients were then divided into subgroups according to MELD score (≥ 30 or < 30), MELD-Na score (≥ 30 or < 30), CTP classification B or C, pre-existing chronic liver diseases (cirrhosis or hepatitis), and HBV DNA level (HBV DNA ≥ 156,000 IU/mL or HBV DNA < 156,000 IU/mL [156,000 = median HBV DNA level]). The 30- and 90-day survival rates of the subgroups based on TSH cut-off value were examined. With the exception of the CTP classification B subgroup, 30-day and 90-day survival rates were increased in all subgroups with higher TSH level (log-rank test *P* < 0.001) (Additional file 2: Fig. S1, Additional file 3: fig. S2, Additional file 4: fig. S3, Additional file 5: fig. S4, Additional file 6: fig. S5).

### HRs for 30-day and 90-day survival according to TSH cut-off values

As shown in Tables 3, the MELD score-adjusted HRs (Group 2 vs. Group 1) for 30-day and 90-day survival were 0.542 (95% CI: 0.457–0.642, *P* < 0.001) and 0.646 (95% CI: 0.564–0.740, *P* < 0.001), respectively. After adjusting for age and other risk factors, the higher level of TSH remained associated with 30-day survival (HR = 0.602, 95% CI: 0.502–0.721, *P* < 0.001) and 90-day survival (HR = 0.704, 95% CI, 0.609–0.814, *P* < 0.001).

**Table 3:** HRs for 30-day and 90-day survival according to TSH cut-off values

	30-day survival ( TSH ≥ 0.261 vs. TSH < 0.261)			90-day survival (TSH ≥ 0.261 vs. TSH < 0.261)		
	HR	95%CI	*P* value	HR	95%CI	*P* value
Unadjusted	0.414	0.351–0.490	< 0.001	0.526	0.460–0.601	< 0.001
MELD score-adjusted	0.542	0.457–0.642	< 0.001	0.646	0.564–0.740	< 0.001
MELD-Na score-adjusted	0.505	0.426–0.598	< 0.001	0.614	0.536–0.703	< 0.001
CTP score-adjusted	0.455	0.385–0.539	< 0.001	0.568	0.496–0.650	< 0.001
Multiple Cox analysis risk factors adjusted^†^	0.602	0.502–0.721	< 0.001	0.704	0. 609–0.814	< 0.001

^†^ Obtained from Cox proportional hazard regression models (a) 30-day survival: adjusted for age, white blood cell count, glutamic-oxaloacetic transaminase, albumin, total bilirubin, INR, international normalized ratio, serum creatinine, alpha fetal protein, hepatitis B virus (> 156,000 vs. < 156,000), pre-existing chronic liver diseases (cirrhosis vs. chronic hepatitis), hepatorenal syndrome (yes vs. no) and hepatic encephalopathy(grade 3 vs. grade 1–2 vs. none); (b) 90-day survival: adjusted for age, white blood cell count, glutamic-oxaloacetic transaminase, total bilirubin, INR, international normalized ratio, serum creatinine, alpha fetal protein, hepatitis B virus (> 156,000 vs. < 156,000), pre-existing chronic liver diseases (cirrhosis vs. chronic hepatitis), hepatic encephalopathy(grade 3 vs. grade 1–2 vs. none), gastrointestinal bleeding (yes vs. no) and infection (yes vs. no)

*ACLF* Acute-on-chronic liver failure; *CTP* Child-Turcotte-Pugh; *MELD* Model for End-Stage Liver Disease; *MELD-Na* Model for End-Stage Liver Disease with the addition of the Na level; *TSH* thyroid-stimulation hormone

## Discussion

Thyroid hormones are vital mediators of multiple physiological processes. The liver is a target organ of thyroid hormones, and also plays an important role in the metabolism of the hormones [11]. Studies have demonstrated that thyroid dysfunction is associated with various conditions such as heart failure [12], cerebrovascular disorders [13], kidney disease [14], and liver cirrhosis [15]. However, it is still unclear if TSH level holds prognostic value for patients with HBV-related ACLF [15, 24, 25]. In this large-scale observational study of 1,862 patients with HBV-related ACLF, we found that TSH levels before treatment were correlated with reduced 30-day and 90-day survival, and that TSH level was an independent predictor of 30-day and 90-day outcomes. After adjusting for MELD score, MELD-Na score or other risk factors, the higher level of TSH still had protective effect on both 30-day and 90-day survival.

MELD score is commonly used to determine if patients with ACLF are candidates for transplantation [6]. However, it primarily uses the indexes of TBil, INR, and serum Cr, and studies have shown that it does not fully reflect the severity of liver failure [27]. In recent years, the CLIF-C OF score, the CLIF-C ACLF score, and the COSSH ACLF score have been proposed as measures to predict the outcomes of patients with ACLF. The 3 methods evaluate the patients’ condition from the aspect of multiple organ dysfunction. Nevertheless, abnormal liver function can also lead to disorders of the endocrine system, including thyroid dysfunction, and current models do not include thyroid hormone levels in the scoring systems. The results of this study suggest that TSH is an independent factor that can predict the 30-day and 90-day survival of patients with HBV-related ACLF, and thus provides an important complementary marker for establishing prognosis. The results also showed that except for subgroup of CTP classification B patients, 30-day and 90-day survival rates were positively correlated with TSH concentration. There are some possible reasons why the finding did not apply to CTP classification B patients. 1) Risk factors were not adjusted at baseline, which may lead to bias. 2) The number of CTP classification B patients was relatively small.

Prior studies have provided conflicting results with respect to the association between TSH level and the outcomes of patients with ACLF. Tas et al. [15] reported that TSH level was not related to the outcomes of patients with cirrhosis requiring intensive care unit (ICU) admission, a finding that is not consistent with the results of our study. We speculate that the different findings may be due to the different causes of liver damage. Systemic inflammation and inflammatory factor storm caused by immune imbalance are considered to be the main mechanisms of ACLF [28]. On the other hand, cirrhosis is due to the formation of pseudolobules caused by liver fibrosis, which leads to portal hypertension and other related complications [15]. Studies by Wu D et al. [22] and Wu Y et al. [23] indicated that TSH level was an independent prognostic factor for patients with HBV-related ACLF. Although the results of the 2 studies are consistent with ours, there are differences between the studies. The study by Wu Y et al. only included 75 patients with ACLF, and common factors (e.g., TB, INR, WBC count, HE) that affect the prognosis of ACLF patients were not adjusted for in the Cox risk regression analysis. In the study by Wu D et al., the length of follow-up was only 30 days.

The mechanism by which TSH is associated with the survival of patients with HBV-related ACLF is unclear. One possible reason is the suppression of hypothalamic-pituitary-thyroid axis in ACLF patients, which is considered to be related to the influence of inflammatory factors (e.g., IL-6, IL-1, TNF-α) [24, 29]; and immune damage caused by inflammatory factor storm is the main pathogenic mechanism of ACLF [28]. Another possible reason for the association is that cerebral edema due to HE is thought to impact the function of hypothalamic-pituitary-thyroid axis, thus affecting the secretion of TSH [30]. Thirdly, thyroid hormones possess the ability to induce the proliferation of hepatocytes [31], and TSH promotes the secretion of thyroid hormones. A higher level of TSH may indirectly indicate the regeneration of hepatocytes, which thereby results in a positive correlation between the survival rate of ACLF patients and TSH level. Fourthly, studies showed that TSHR is present and functional in hepatocytes and NK cells [16, 17]. And TSH can influence glucose and lipid homeostasis in hepatocytes via TSHR directly [32, 33]. In addition, Wang X et al. showed that TSH plays an essential role in mitochondrial oxidative stress in the liver via TSHR [21]. Oxidative stress has been reported to be the collective pathophysiological mechanism of many liver diseases. We speculated that the direct action between TSH and hepatocyte via TSHR and/or the potential immunomodulatory ability of TSH might be two of the important mechanisms. But it still needs further studies to confirm.

There are some limitations to this study that should be considered. First, 90-days is a relatively short follow-up period, and a longer observation period can help confirm the predictive value of TSH level. Second, TSH level was only measured once. Measurements at multiple time points may help to confirm the predictive value of TSH level, and improve the accuracy. Third, 579 were excluded due to incomplete data and/or lost to follow-up. This may cause selective bias to the results of the study. Fourth, this is a retrospective study and subject to all standard limitations associated with this format.

## Conclusions

In summary, the results of this study suggest that TSH level significantly correlate with the 30-day and 90-day survival of patients with HBV-related ACLF. Measurement of TSH is widely available, and should be considered for routine testing in ACLF patients. The results of this study need to be validated in a multicenter and prospective study.

## Supplementary Information

**Additional file 1. Table S1** Univariate Cox proportional hazards regression analysis

**Additional file 2. Figure S1** Kaplan-Meier curves of HBV-related ACLF patients stratified by MELD score. (Group 1: <0.261 µIU/mL, Group 2: ≥0.261 µIU/mL; A, B: MELD score <30; C, D: MELD score≥30) ACLF, acute-on-chronic liver failure; HBV, hepatitis B virus; MELD, Model for End-Stage Liver Disease; TSH, thyroid-stimulation hormone

**Additional file 3. Figure S2** Kaplan-Meier curves of HBV-related ACLF patients stratified by MELD-Na score. (Group 1: <0.261 µIU/mL, Group 2: ≥0.261 µIU/mL; A, B: MELD-Na score <30; C, D: MELD-Na score≥30) ACLF, acute-on-chronic liver failure; HBV, hepatitis B virus; MELD-Na, Model for End-Stage Liver Disease with the addition of the Na level; TSH, thyroid-stimulation hormone

**Additional file 4. Figure S3** Kaplan-Meier curves of HBV-related ACLF patients stratified by CTP classification. (Group 1: <0.261 µIU/mL, Group 2: ≥0.261 µIU/mL; A, B: CTP classification B; C, D: CTP classification C) ACLF, acute-on-chronic liver failure; CTP, Child-Turcotte-Pugh; HBV, hepatitis B virus; TSH, thyroid-stimulation hormone

**Additional file 5. Figure S4** Kaplan-Meier curves of HBV-related ACLF patients stratified by pre-existing chronic liver diseases. (Group 1: <0.261 µIU/mL, Group 2: ≥0.261 µIU/mL; A, B: hepatitis patients; C, D: patients with cirrhosis) ACLF, acute-on-chronic liver failure; HBV, hepatitis B virus; TSH, thyroid-stimulation hormone

**Additional file 6. Figure S5** Kaplan-Meier curves of HBV-related ACLF patients stratified by HBV DNA. (Group 1: <0.261 µIU/mL, Group 2: ≥0.261 µIU/mL; A, B: HBV DNA <156000IU/mL; C, D: HBV DNA≥156000IU/mL) ACLF, acute-on-chronic liver failure; HBV, hepatitis B virus; TSH, thyroid-stimulation hormone

## Data Availability

The datasets used and/or analyzed during the current study are available from the corresponding author on reasonable request.

## Abbreviations

ACLF: Acute-on-chronic liver failure
AFP: Alpha fetoprotein
ALB: Albumin
ALT: Alanine aminotransferase
AST: Aspartate aminotransferase
AUROC: Area under the ROC curve
CI: Confidence interval
CLIF-C OF: Chronic liver failure consortium organ failure
COSSH: Chinese group on the study of severe hepatitis B
Cr: Creatinine
CTP: Child-Turcotte-Pugh
EASL: European association for the study of the liver
GB: Gastrointestinal bleeding
Hb: Hemoglobin
HBeAb: HBV e antibody
HBeAg: HBV e antigen
HBV: Hepatitis B virus
HCV: Hepatitis C virus
HE: Hepatic encephalopathy
HR: Hazard ratio
HRS: Hepatorenal syndrome
ICU: Intensive care unit
INR: International normalized ratio
IQR: Interquartile range
MELD: Model for end-stage liver disease
MELD-Na: Model for end-stage liver disease with the addition of the Na level
Na: Serum sodium
PLT: Platelet
TBil: Total bilirubin
TSH: Thyroid-stimulation hormone
TSHR: Thyroid-stimulation hormone receptor
WBC: White blood cell
