# Supplementary material for: The impact of serum thyroid-stimulation hormone levels on the outcome of hepatitis B virus related acute-on-chronic liver failure: an observational study
Source: BMC Gastroenterol. 2022 Jul 7;22:330. doi: 10.1186/s12876-022-02406-7 (PMC9260984; doi:10.1186/s12876-022-02406-7)
Supplement: Supplementary file 1 — Additional file 1. Table S1 Univariate Cox proportional hazards regression analysis [file 12876_2022_2406_MOESM1_ESM.docx]

1. **Supplementary figure legend**

**Supplementary Figure 1** Kaplan-Meier curves of HBV-related ACLF patients stratified by MELD score. (Group 1: <0.261 µIU/mL, Group 2: ≥0.261 µIU/mL; A, B: MELD score <30; C, D: MELD score≥30) ACLF, acute-on-chronic liver failure; HBV, hepatitis B virus; MELD, Model for End-Stage Liver Disease; TSH, thyroid-stimulation hormone.

**Supplementary Figure 2** Kaplan-Meier curves of HBV-related ACLF patients stratified by MELD-Na score. (Group 1: <0.261 µIU/mL, Group 2: ≥0.261 µIU/mL; A, B: MELD-Na score <30; C, D: MELD-Na score≥30) ACLF, acute-on-chronic liver failure; HBV, hepatitis B virus; MELD-Na, Model for End-Stage Liver Disease with the addition of the Na level; TSH, thyroid-stimulation hormone.

**Supplementary Figure 3** Kaplan-Meier curves of HBV-related ACLF patients stratified by CTP classification. (Group 1: <0.261 µIU/mL, Group 2: ≥0.261 µIU/mL; A, B: CTP classification B; C, D: CTP classification C) ACLF, acute-on-chronic liver failure; CTP, Child-Turcotte-Pugh; HBV, hepatitis B virus; TSH, thyroid-stimulation hormone.

**Supplementary Figure 4** Kaplan-Meier curves of HBV-related ACLF patients stratified by pre-existing chronic liver diseases. (Group 1: <0.261 µIU/mL, Group 2: ≥0.261 µIU/mL; A, B: hepatitis patients; C, D: patients with cirrhosis) ACLF, acute-on-chronic liver failure; HBV, hepatitis B virus; TSH, thyroid-stimulation hormone.

**Supplementary Figure 5** Kaplan-Meier curves of HBV-related ACLF patients stratified by HBV DNA. (Group 1: <0.261 µIU/mL, Group 2: ≥0.261 µIU/mL; A, B: HBV DNA <156000IU/mL; C, D: HBV DNA≥156000IU/mL) ACLF, acute-on-chronic liver failure; HBV, hepatitis B virus; TSH, thyroid-stimulation hormone.

1. **Supplementary table**

| **Supplementary Table 1. Univariate Cox proportional hazards regression analysis** | | | | | | | |
| --- | --- | --- | --- | --- | --- | --- | --- |
|  | **30 days survival** | | |  | **90 days survival** | | |
| **Variable** | **HR** | **95%CI** | **P** |  | **HR** | **95%CI** | **P** |
| **Age,y** | 1.034 | 1.028-1.041 | <0.001 |  | 1.037 | 1.031-1.042 | <0.001 |
| **Sex (Female vs. Male)** | 1.319 | 1.034-1.682 | 0.026 |  | 1.150 | 0.934-1.415 | 0.187 |
| **WBC (×10^9^/L)** | 1.083 | 1.066-1.100 | <0.001 |  | 1.062 | 1.046-1.078 | <0.001 |
| **Hb (g/L)** | 0.994 | 0.990-0.998 | 0.002 |  | 0.993 | 0.990-0.996 | <0.001 |
| **PLT (×10^9^/L)** | 0.998 | 0.997-1.000 | 0.008 |  | 0.996 | 0.995-0.998 | <0.001 |
| **ALT (U/L)** | 1.000 | 1.000-1.000 | 0.011 |  | 1.000 | 1.000-1.000 | 0.955 |
| **AST (U/L)** | 1.000 | 1.000-1.000 | <0.001 |  | 1.000 | 1.000-1.000 | 0.001 |
| **ALB (g/L)** | 0.945 | 0.927-0.963 | <0.001 |  | 0.999 | 0.994-1.004 | 0.647 |
| **GLB (g/L)** | 0.996 | 0.984-1.007 | 0.461 |  | 1.007 | 0.998-1.017 | 0.117 |
| **TBil (µmol/L)** | 1.002 | 1.002-1.003 | <0.001 |  | 1.002 | 1.002-1.003 | <0.001 |
| **INR** | 1.085 | 1.071-1.099 | <0.001 |  | 1.080 | 1.067-1.094 | <0.001 |
| **Na (mmol/L)** | 0.982 | 0.975-0.988 | <0.001 |  | 0.981 | 0.976-0.986 | <0.001 |
| **Cr (µmol/L)** | 1.004 | 1.004-1.005 | <0.001 |  | 1.004 | 1.003-1.005 | <0.001 |
| **AFP (ng/mL)** | 0.997 | 0.996-0.998 | <0.001 |  | 0.998 | 0.997-0.998 | <0.001 |
| **HBeAg(Positive vs. Negative)** | 0.781 | 0.649-0.940 | 0.009 |  | 0.739 | 0.635-0.859 | <0.001 |
| **HBV DNA (IU/mL, >156000 vs. <156000)** | 1.367 | 1.157-1.615 | <0.001 |  | 1.177 | 1.029-1.346 | 0.017 |
| **TSH(µIU/mL)** | 0.521 | 0.441-0.615 | <0.001 |  | 0.648 | 0.576-0.729 | <0.001 |
| **Pre-existing chronic liver diseases (Cirrhosis vs. Chronic hepatitis)** | 1.642 | 1.372-1.964 | <0.001 |  | 2.015 | 1.737-2.339 | <0.001 |
| **Ascite (Grade 3 vs. Grade 1-2 vs. None)** | 1.081 | 0.956-1.222 | 0.214 |  | 1.197 | 1.083-1.322 | <0.001 |
| **Hepatorenal Syndrome (Yes vs. No)** | 4.343 | 3.399-5.719 | <0.001 |  | 3.964 | 3.078-5.108 | <0.001 |
| **Hepatic Encephalopathy (Grade 3 vs. Grade 1-2 vs. None)** | 3.170 | 2.791-3.601 | <0.001 |  | 2.615 | 2.336-2.928 | <0.001 |
| **Gastrointestinal Bleeding (Yes vs. No)** | 3.126 | 1.928-5.067 | <0.001 |  | 3.043 | 1.972-4.696 | <0.001 |
| **Infection (Yes vs. No)** | 1.957 | 1.579-2.426 | <0.001 |  | 1.907 | 1.611-2.258 | <0.001 |

AFP, alpha fetal protein; ALB, albumin; ALT, alanine aminotransferase; AST, glutamic-oxaloacetic transaminase; Cr, serum creatinine; CI, confidence interval; GLB, globulin; Hb, hemoglobin; HBeAg, hepatitis B e antigen; HBV, hepatitis B virus; HR, hazard ratio; INR, international normalized ratio; Na, serum sodiun; PLT, platelet; TBil, total bilirubin; TSH, thyroid-stimulation hormone; WBC, white blood cell.
