# Supplementary figures and images for: The impact of serum thyroid-stimulation hormone levels on the outcome of hepatitis B virus related acute-on-chronic liver failure: an observational study
Source: BMC Gastroenterol. 2022 Jul 7;22:330. doi: 10.1186/s12876-022-02406-7 (PMC9260984; doi:10.1186/s12876-022-02406-7)

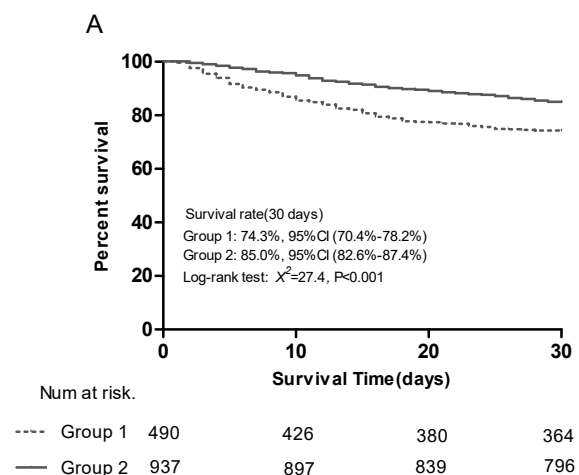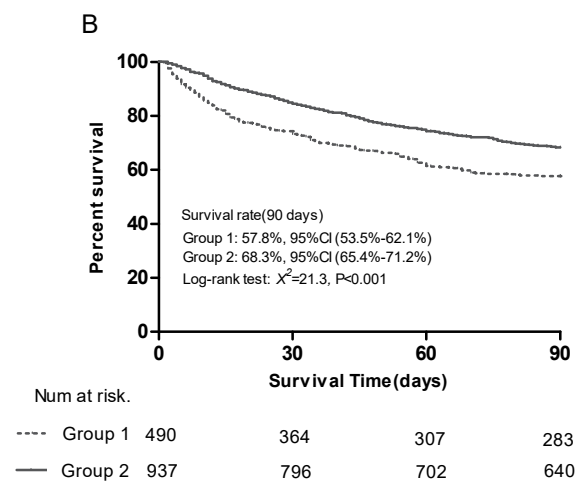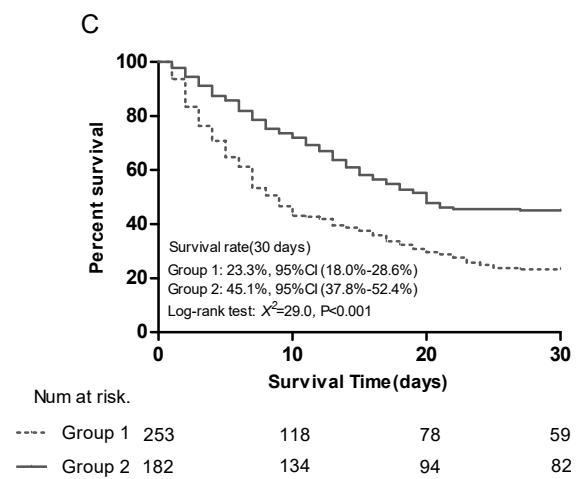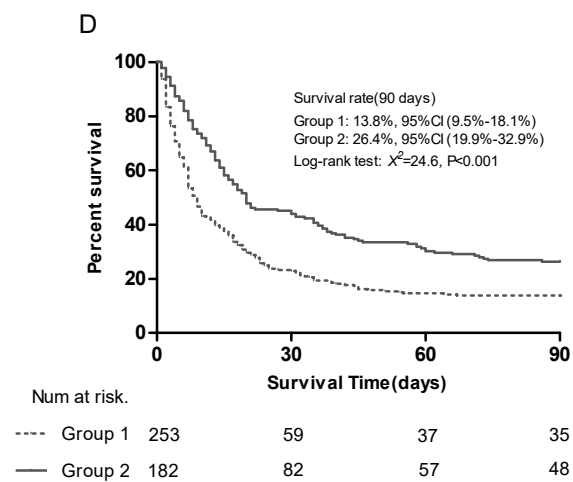

Supplement: Supplementary file 2 — Additional file 2. Figure S1 Kaplan-Meier curves of HBV-related ACLF patients stratified by MELD score. (Group 1: <0.261 µIU/mL, Group 2: ≥0.261 µIU/mL; A, B: MELD score <30; C, D: MELD score≥30) ACLF, acute-on-chronic liver failure; HBV, hepatitis B virus; MELD, Model for End-Stage Liver Disease; TSH, thyroid-stimulation hormone [file 12876_2022_2406_MOESM2_ESM.pdf]

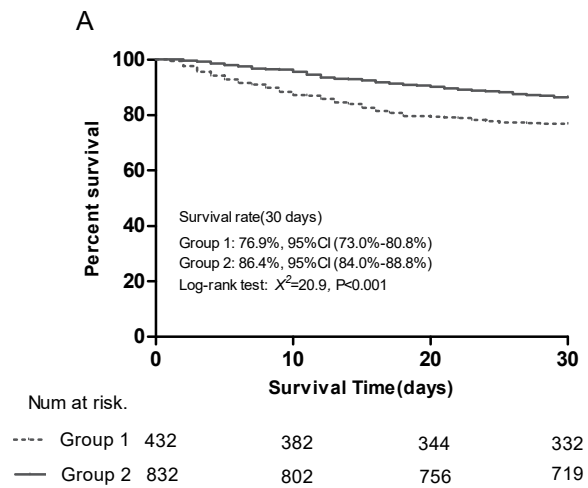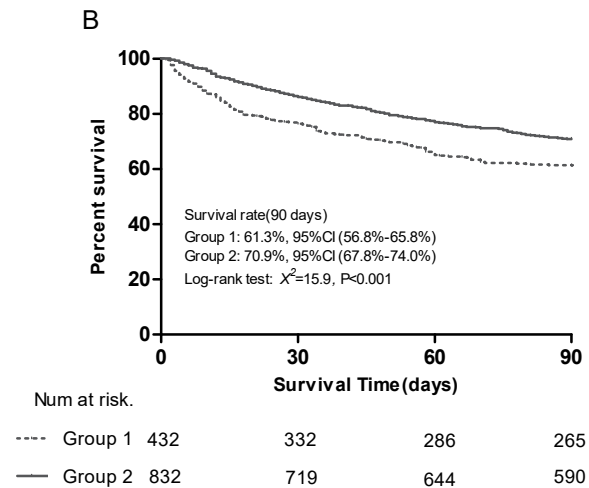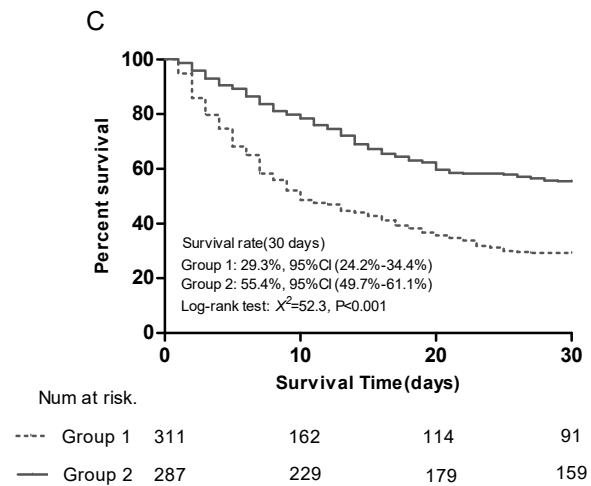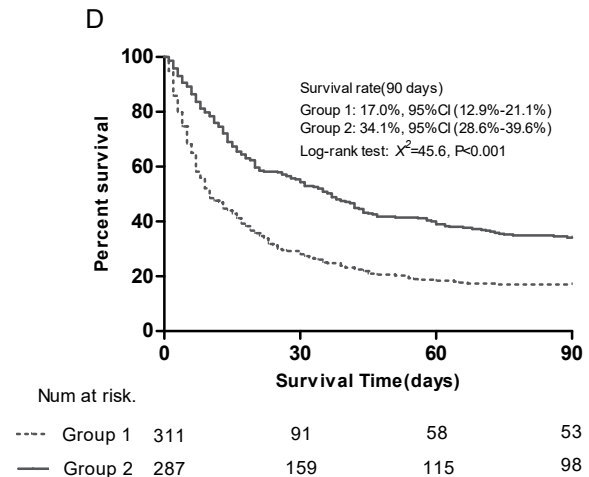

Supplement: Supplementary file 3 — Additional file 3. Figure S2 Kaplan-Meier curves of HBV-related ACLF patients stratified by MELD-Na score. (Group 1: <0.261 µIU/mL, Group 2: ≥0.261 µIU/mL; A, B: MELD-Na score <30; C, D: MELD-Na score≥30) ACLF, acute-on-chronic liver failure; HBV, hepatitis B virus; MELD-Na, Model for End-Stage Liver Disease with the addition of the Na level; TSH, thyroid-stimulation hormone [file 12876_2022_2406_MOESM3_ESM.pdf]

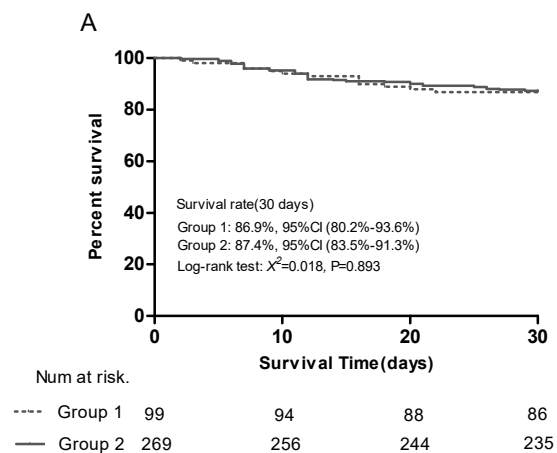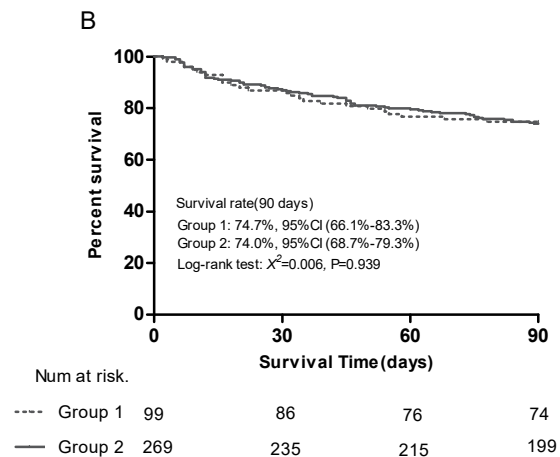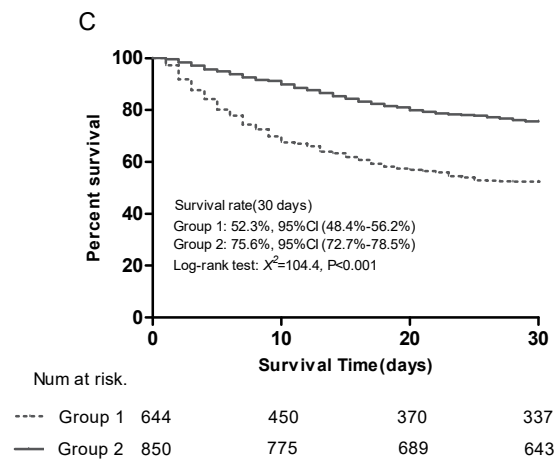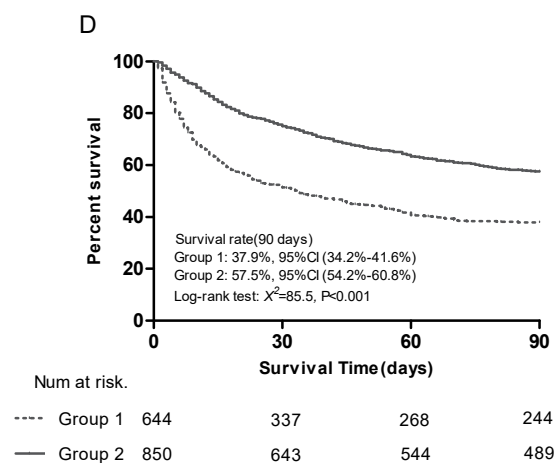

Supplement: Supplementary file 4 — Additional file 4. Figure S3 Kaplan-Meier curves of HBV-related ACLF patients stratified by CTP classification. (Group 1: <0.261 µIU/mL, Group 2: ≥0.261 µIU/mL; A, B: CTP classification B; C, D: CTP classification C) ACLF, acute-on-chronic liver failure; CTP, Child-Turcotte-Pugh; HBV, hepatitis B virus; TSH, thyroid-stimulation hormone [file 12876_2022_2406_MOESM4_ESM.pdf]

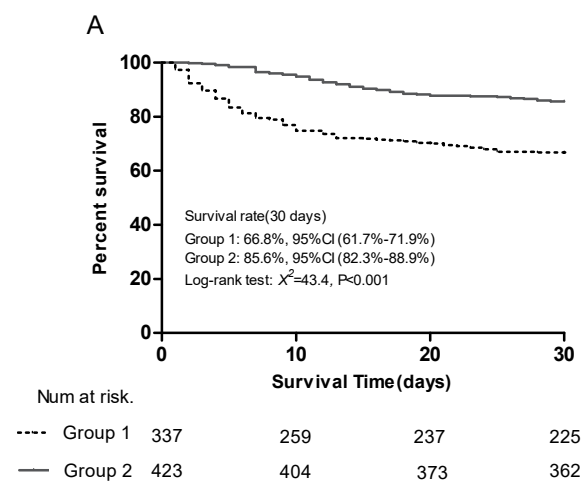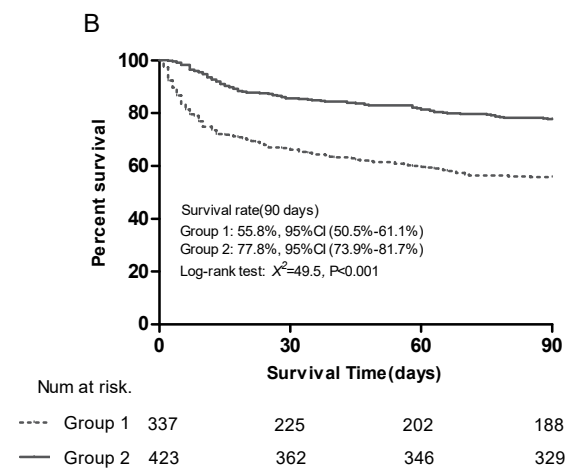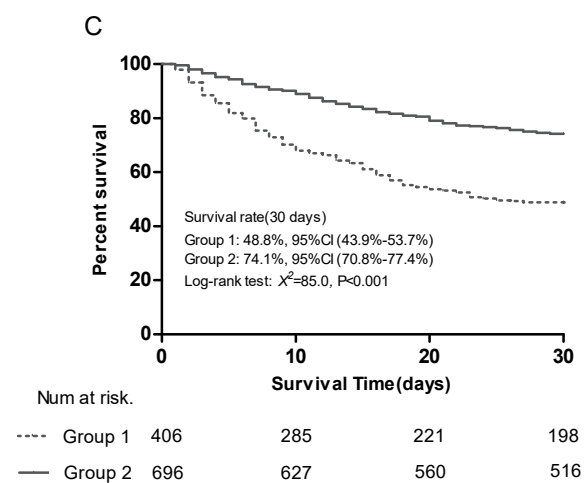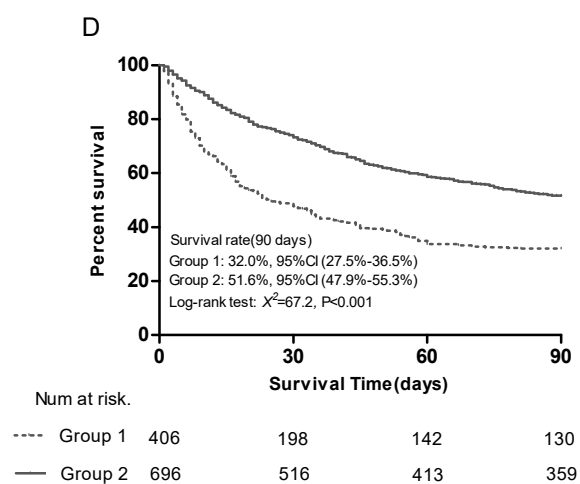

Supplement: Supplementary file 5 — Additional file 5. Figure S4 Kaplan-Meier curves of HBV-related ACLF patients stratified by pre-existing chronic liver diseases. (Group 1: <0.261 µIU/mL, Group 2: ≥0.261 µIU/mL; A, B: hepatitis patients; C, D: patients with cirrhosis) ACLF, acute-on-chronic liver failure; HBV, hepatitis B virus; TSH, thyroid-stimulation hormone [file 12876_2022_2406_MOESM5_ESM.pdf]

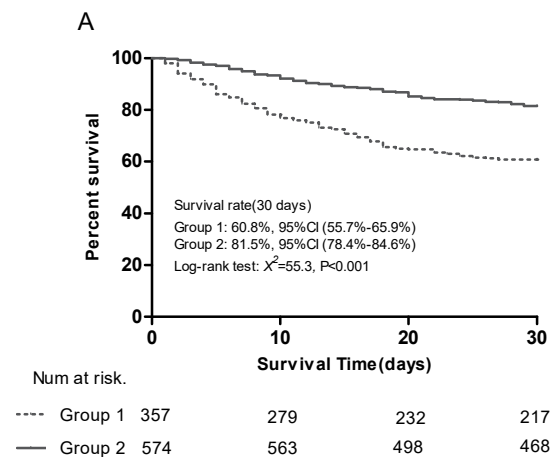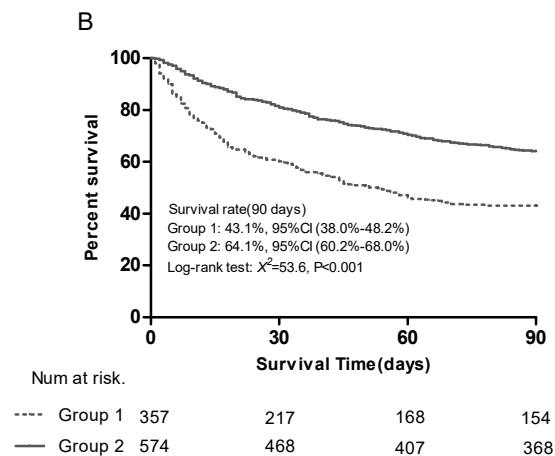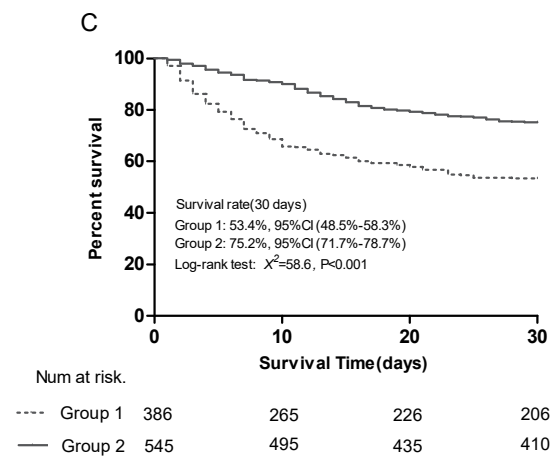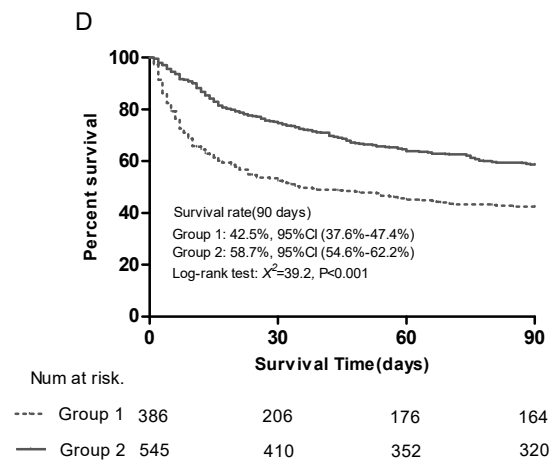

Supplement: Supplementary file 6 — Additional file 6. Figure S5 Kaplan-Meier curves of HBV-related ACLF patients stratified by HBV DNA. (Group 1: <0.261 µIU/mL, Group 2: ≥0.261 µIU/mL; A, B: HBV DNA <156000IU/mL; C, D: HBV DNA≥156000IU/mL) ACLF, acute-on-chronic liver failure; HBV, hepatitis B virus; TSH, thyroid-stimulation hormone [file 12876_2022_2406_MOESM6_ESM.pdf]
